# On Reporting Cases

**Published:** 1889-12

**Authors:** 


### ON REPORTING CASES.

In the *Allg. Hom. Zeitung*, No. 24, Dr. Paul Lutze reports a case of periostitis, which truly demonstrates how, from wrong premises, only false conclusions must follow, and his teachings of the case allow, therefore, many objections. He says:

"I publish this case for two reasons: to show that sometimes the remedy does not cure which corresponds to the totality of the symptoms, but rather the keynote remedy must be selected; and second, to lead the attention of younger physicians to a remedy not often used. On the 30th of January I was called to a lady and found her in a sitting position, the foot resting on a chair, as walking or standing caused severe pain. I found on the tibia a red hot spot of the size of a hand, very sensitive to the touch, and offering small inequalities to the fingers. Mercurius sol. always did well for me in periostitis, and I prescribed Mercur.$^{30}$, ten globules in water, to take a sip every four hours. February 2d.—The inflamed spot paler and less painful, but the inflammation spreads upward and downward. Mercur. continued. February 7th.—Inflammation about the same, with swelling around the malleoli of the affected limb. Apis.$^{30}$ twice daily for three days and an interval of two days. February 17th.—The swelling is gone, the inflammation pales, but she complains of a painful drawing and of stiffness in the joints of both knees and feet in afternoon and evening. Bryonia$^{30}$, to be taken in the same manner. Next report was that the drawing pains were removed, that the thickening on the tibia still persisted, that it did not look as much inflamed; but on the condyle of the right knee-joint periosteal swellings had arisen, painful by motion and pressure. Mercur.$^{30}$ brought no relief this time; in fact, pains were worse in the evening, and even a return to Bryonia gave no relief. Among the remedies having "pains increased by motion," I also found Ledum, and though it was aggravation from the heat of the bed, which may also mean at the time of going to bed, my experience taught me, that in rare cases the simile may succeed where the simillimum fails. I prescribed Ledum.$^{30}$, a swallow every four hours, and in a few days the patient remarked that all pains quickly disappeared; the periosteal thickenings were nearly gone, and walking or standing nearly painless. March 30th she complained only of some stitching pains when standing, which were quickly removed with Mezereum$^{30}$.

Dr. Lutze fails to give us an anamnesis of the case, and we do not know what sort of a woman he had to deal with, and what was the cause of the periostitis. Was it of traumatic, syphilitic, or scrofulous origin? Was it painful to the touch, even when resting, or did only the motion irritate the sore? In too many cases reported in the journals, we find the same omission, and thus much of its value is lost. We cannot believe that Dr. Lutze prescribed for pathological names, and still he acknowledges that he gave Mercur. *ex usu in morbo*, and, as usual, as it covered only some symptoms, it acted like every other palliative but did not cure the case. Lutze jumped then to Apis on account of some œdematous swelling around the maleoli. Again, a mere palliative prescription which did not reach the root of the evil, for he changes again to Bryonia, on account of some stiffness in knee and ankle-joints of both feet, afternoon and evening, and as now appeared some thickening in the condyle of the right knee-joint, he returned to Mercur., but failed to give relief, and Bryonia was given on account of the stereotyped symptom, < by motion and in the evening. In his despair he hit on Ledum, as it has also < on motion, *but it cured*, because it is the remedy for gouty pains, especially in the joints of feet and hands, shifting from place to place, involving also fibrous parts in various places; pains in bones of ankles, in knees, toes; < toward evening, till lying down; > at night; pains sticking, tearing, shifting; swelling about ankles, with pain in ankles when stepping. Ledum is a cold remedy and has hardly any febrile symptoms, while Bryonia and Mercur. are full of them. We thus see how many symptoms were fully covered with the Marsh tea, and that it was at least a close simile, taking the gouty constitution of the patient as a guide. He finished the case with Mezereum, on account of some stitching pains when standing. Might we not ask whether Mezereum was not from the start the simillimum to the case? Hering recommended it for swollen, inflamed bones, especially shafts of cylindrical bones, pain in the periosteum of long bones, especially the tibiæ, < at night in bed, the least touch unbearable, and < in damp weather, with general chill and thirst; boring, drawing pains in ankles; short drawing or darting pains, now here, now there, after which a constant soreness remains; itching, burning, shifting pains, with increase of animal heat. Who of us can always find the simillimum? But we must differ from Dr. Lutze, when he holds up such a case as a bright example for the student to follow. We may fail, but we need not glory about it. S. L.

